# Fabrication and Photocatalytic Activity of Ag_3_PO_4_/T-ZnOw Heterostructures

**DOI:** 10.1186/s11671-020-03363-4

**Published:** 2020-06-15

**Authors:** Jianke Tang, Rongqian Meng, Qi Wang, Shengjian Zhang, Qiaoling Li

**Affiliations:** 1 School of Chemical Engineering and Technology, North University of China, Taiyuan, 030051 Shanxi People’s Republic of China; 2 Department of Chemistry and Chemical Engineering, Taiyuan Institute of Technology, Taiyuan, 030008 Shanxi People’s Republic of China

**Keywords:** Ag_3_PO_4_/T-ZnOw, Heterostructures, Visible light, Photocatalytic

## Abstract

The Ag_3_PO_4_/tetrapod-like ZnO whisker (T-ZnOw) heterostructures were prepared via a simple precipitation method. The obtained heterostructures were characterized by X-ray diffraction, scanning electron microscopy, transmission electron microscopy, high-resolution transmission electron microscopy, X-ray photoelectron spectroscopy, and UV-Vis diffuse reflectance spectroscopy. The photodegradation activity of Ag_3_PO_4_/T-ZnOw was evaluated by the degradation of Rhodamine B (RhB) under visible light irradiation. When the molar ratio of Ag_3_PO_4_ to T-ZnOw was 10% (Ag_3_PO_4_/T-ZnOw-2), the highest degradation efficiency (92.9%) could be achieved among the heterostructures. The photodegradation rate constant of Ag_3_PO_4_/T-ZnOw-2 (0.05179 min^−1^) was 3.59 times that of T-ZnOw (0.01444 min^−1^). Besides, the Ag_3_PO_4_/T-ZnOw-2 photocatalyst still possessed a degradation efficiency of 77.8% after four successive cycles. The Ag_3_PO_4_/T-ZnOw-2 catalyst had much higher photocatalytic activity than pure T-ZnOw and better stability and reusability than pure Ag_3_PO_4_. The effect of different scavengers on degradation efficiency was investigated, and the possible photocatalytic mechanism of the Ag_3_PO_4_/T-ZnOw photocatalyst was also put forward.

## Introduction

Dye wastewater pollution from the textile industries has been a major environmental issue in recent decades due to non-biodegradability and potential carcinogenicity. Currently, the researchers have explored various techniques to handle the pollutants in wastewater. Semiconductor photocatalysis technology has been considered as an effective way for the purification of polluted water [1–6]. Zinc oxide (ZnO), an environmentally friendly photocatalytic material, has been extensively studied due to its features of low cost, high controllability, and thermal and chemical stability [7–11]. Unfortunately, the wide bandgap (3.37 eV) of ZnO restrains its large-scale practical applications in visible light [12]. Furthermore, the low separation rate of the photogenerated electron-hole pairs also limits the photocatalytic performance of ZnO. For the modification of ZnO photocatalysts, an effective strategy is to shift the absorption band from ultraviolet to visible light range, enabling absorption of more energy from solar irradiation and enhancing the utilization of solar light [13]. It is generally known that coupling ZnO with narrow bandgap semiconductors can be an effective way to absorb more energy from the solar irradiation and enhance the photocatalytic activity. Besides, the formation of heterostructures with a properly matched energy gap can also enhance the separation of charge carriers in photocatalysts. For instance, AgBr/ZnO [14], ZnO/BiOI [15], ZnO/AgI [16], Ag_3_VO_4_/ZnO [17], Ag_2_CO_3_/ZnO [18], Ag_2_O/ZnO [19], and BiVO_4_/ZnO [20] have been reported.

Recently, the silver orthophosphate (Ag_3_PO_4_) has attracted considerable attention as a promising coupling material due to a narrow band gap (about 2.4 eV) [21], which showed high photodegradation efficiency of organic pollutions in aqueous solution under visible light [22–25]. However, the Ag_3_PO_4_ can be reduced to Ag^0^ during the photocatalytic process due to the photocorrosion of the photogenerated electrons under visible light irradiation, which may decrease the structural stability and reusability, and strongly limit the long-term application for water treatment [23, 26–28]. Besides, the use of a large amount of expensive silver-containing material in the photocatalytic system strongly increased operating costs. As previously reported, the stability of Ag_3_PO_4_ can be enhanced by the preparation of composites over a supporting material of matched electronic structure and the composites showed excellent photocatalytic performance at the same time [27, 29–31].

In this work, we deposited Ag_3_PO_4_ particles on T-ZnOw surfaces by a facile in situ deposition method at room temperature. In the Ag_3_PO_4_/T-ZnOw composites, T-ZnOw works as a substrate, which has unique shape and structure, low density of native defects, and large specific surface areas [32–35]. The photocatalytic activities of the Ag_3_PO_4_/T-ZnOw composites were investigated by decomposing RhB under the irradiation of visible light, and the stability was also determined. Furthermore, the possible photocatalytic mechanism was also discussed in detail.

## Methods

### Materials

T-ZnOw was obtained from Chengdu Crystrealm Co. Ltd. (Chengdu, China). Silver nitrate (AgNO_3_, > 99.8%) was purchased from Tianjin Fengchuan Chemical Reagent Co. Ltd. (Tianjin, China). Sodium phosphate dibasic dodecahydrate (Na_2_HPO_4_·12H_2_O, 99.0%) and benzoquinone (BQ) were purchased from Aladdin Reagents Company (Shanghai, China). RhB was provided by Macklin Biochemical Company (Shanghai, China). Isopropyl alcohol (IPA) was obtained from Tianjin Kemiou Chemical Co. Ltd. (Tianjin, China). Ethylenediaminetetraacetic acid disodium salt (EDTA-2Na) was purchased from Tianjin Shentai Chemical Industry Co. Ltd. (Tianjin, China). Absolute ethanol was obtained from Sinopharm Chemical Reagent Co. Ltd. (Shanghai, China). Deionized water with resistivity of 18.2 MΩ cm was used in all cases from an ULUPURE water purification system (Chengdu, China).

### Preparation of Photocatalysts

An in situ precipitation method was employed to prepare Ag_3_PO_4_/T-ZnOw composites, and the molar ratios of Ag_3_PO_4_ to T-ZnOw were 5%, 10%, and 15%, respectively. The products were marked as Ag_3_PO_4_/T-ZnOw-1, Ag_3_PO_4_/T-ZnOw-2, and Ag_3_PO_4_/T-ZnOw-3, respectively. For instance, for the Ag_3_PO_4_/T-ZnOw-2 sample, 0.1 g T-ZnOw and 0.0440 g Na_2_HPO_4_·12H_2_O were dispersed into 100 mL deionized water by ultrasound and then magnetic stirred. Next, 0.0626 g AgNO_3_ dissolved in 50 mL of deionized water was slowly added to the above suspension by syringe fixed on the injection pump under magnetically stirring. Subsequently, the reaction system was kept under stirring for 3 h. The Ag_3_PO_4_/T-ZnOw precipitate was collected by centrifugation, washed thoroughly with deionized water and absolute ethanol, and subsequently dried in an oven at 60 °C. For comparison, pure Ag_3_PO_4_ was prepared according to the same process in the absence of T-ZnOw.

### Characterization

The X-ray diffraction (XRD) measurements were carried out on a Rigaku SmartLab diffractometer using Cu K-α as the radiation with a scanning rate of 10°/min. The morphology of the composites was studied by scanning electron microscopy (SEM, JSM-7200F, JEOL, Japan). Energy-dispersive X-ray spectroscopy (EDS) attached to the SEM instrument was used to determine the chemical composition of the product. Transmission electron microscopy (TEM) and high-resolution transmission electron microscopy (HRTEM) images were obtained with a JEM-2100F transmission electron microscope. X-ray photoelectron spectroscopy (XPS) measurements were recorded on Thermo ESCALAB 250XI, and the binding energies (BEs) were calibrated with respect to the C1s peak at 284.6 eV. UV-Vis diffuse reflectance spectra (DRS) measurements were obtained by using a UV-Vis-NIR spectrophotometer (Cary5000, Agilent Technologies, USA) with polytetrafluoroethylene as the reference. Photoluminescence (PL) emission spectra of the samples were measured by F-7000 fluorescence spectrophotometer (Hitachi, Japan) with the excitation wavelength of 355 nm.

### Photocatalysis Experiments

The photocatalysis experiments were tested through photodegradation of RhB under visible light. The experiments were carried out in a 250-mL jacketed glass beaker with cooling water to keep the system temperature constant at room temperature. A 300-W Xenon lamp with a 420-nm cutoff filter provided the visible light. Forty milligrams of Ag_3_PO_4_/T-ZnOw composite was added into 100 mL of 10 mg/L RhB solution. Before turning on the Xenon lamp, the suspensions were stirred in darkness for 30 min to reach an adsorption-desorption equilibrium. The distance between the light source and the surface of the suspensions was 15 cm. Every 10 min, 3 mL suspension was collected and centrifuged to get clear liquid then analyzed on a TU-1901 UV-Vis spectrophotometer (Puxi, China) at 554 nm. The photocatalytic degradation efficiency was calculated as the following formula:

$$ \eta =\left(1-C/{C}_0\right)\times 100\% $$

where *C*_0_ is the initial concentration of RhB and *C* is the concentration of RhB after illumination at time *t*, which varies with the reaction time.

## Results and Discussion

**Fig. 1:**
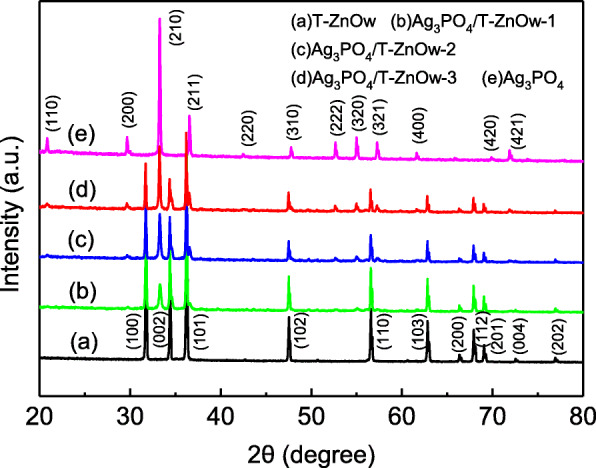
XRD patterns of (a) T-ZnOw, (b) Ag_3_PO_4_/T-ZnOw-1, (c) Ag_3_PO_4_/T-ZnOw-2, (d) Ag_3_PO_4_/T-ZnOw-3, and (e) Ag_3_PO_4_

Figure 1 displayed the XRD patterns of the Ag_3_PO_4_/T-ZnOw composites with different molar ratios of Ag_3_PO_4_, together with those of T-ZnOw and Ag_3_PO_4_. The patterns showed that T-ZnOw was consistent with the standard pattern of ZnO of hexagonal wurtzite phase (JCPDS no. 36-1451) (Fig. 1(a)), while Ag_3_PO_4_ was a crystal of cubic phase (JCPDS no. 06-0505) (Fig. 1(e)). The Ag_3_PO_4_/T-ZnOw composites (Fig. 1(b)–(d)) exhibited a coexistence of both Ag_3_PO_4_ and T-ZnOw. With the molar ratios of Ag_3_PO_4_ increasing, the intensities of the peaks of Ag_3_PO_4_ enhanced markedly, whereas those of T-ZnOw decreased concurrently. The peaks of the Ag_3_PO_4_/T-ZnOw composites were obviously related to T-ZnOw and Ag_3_PO_4_, and no other new crystal phases were found, showing that loading of Ag_3_PO_4_ had not change the crystalline phase of T-ZnOw. These results revealed that Ag_3_PO_4_ particles were successfully deposited on the T-ZnOw surfaces, and Ag_3_PO_4_/T-ZnOw heterostructures were obtained.

**Fig. 2:**
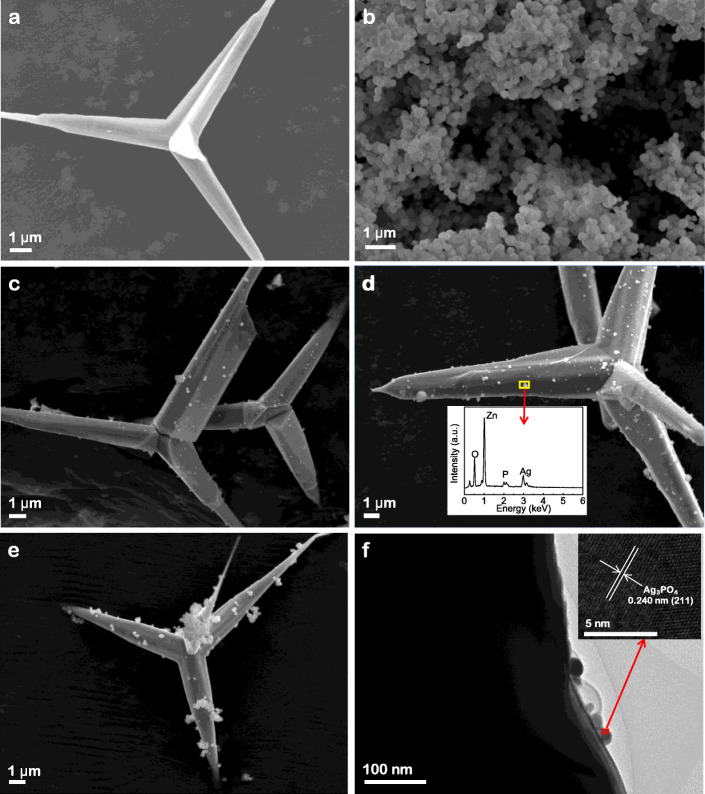
SEM images of **a** T-ZnOw, **b** Ag_3_PO_4_, **c** Ag_3_PO_4_/T-ZnOw-1, **d** Ag_3_PO_4_/T-ZnOw-2 (the inset showed the EDS spectrum of the selected area), **e** Ag_3_PO_4_/T-ZnOw-3, and **f** TEM image of Ag_3_PO_4_/T-ZnOw-2 (the inset showed the HRTEM image of the red rectangle region)

Figure 2 showed the SEM images of T-ZnOw, Ag_3_PO_4_, and the Ag_3_PO_4_/T-ZnOw heterostructures, together with the TEM image and HRTEM image of Ag_3_PO_4_/T-ZnOw-2. T-ZnOw with fairly smooth surface had four legs growing from a common core and extending into the surrounding space. This extension facilitated assembly into a good network with mechanical strength by connecting the legs with each other. Pure Ag_3_PO_4_ exhibited an irregular spherical shape with a diameter of 150–500 nm. The size of T-ZnOw was at micron level, whereas the size of Ag_3_PO_4_ was at nanoscale level. Figure 2c–e displayed the SEM images of Ag_3_PO_4_/T-ZnOw heterostructures. It could be found that nano-sized Ag_3_PO_4_ particles were deposited on the three dimensional (3D) support framework of T-ZnOw. The amount and size of the Ag_3_PO_4_ particles increased with the mole ratios of Ag_3_PO_4_ increasing. When the molar ratio of Ag_3_PO_4_ was 10%, the average diameter of Ag_3_PO_4_ particles was about 150 nm, while further increasing the amount of Ag_3_PO_4_ resulted in the aggregation of Ag_3_PO_4_ particles on the surface of T-ZnOw (Fig. 2e). Figure 2f was the TEM image of the contact interface of the Ag_3_PO_4_/T-ZnOw-2. The nano-sized Ag_3_PO_4_ particles were attached on the surface of T-ZnOw with a good contact. The inset showed the HRTEM image of the red rectangle region of Ag_3_PO_4_/T-ZnOw-2, and the lattice spacing of 0.240 nm corresponds to the (211) crystal plane of Ag_3_PO_4_. The inset of Fig. 2d showed the EDS spectrum corresponding to the rectangle region of the SEM image of the Ag_3_PO_4_/T-ZnOw-2 sample. The sample consisted of four elements, Zn, Ag, O, and P, which was in consistent with the XPS results.

**Fig. 3:**
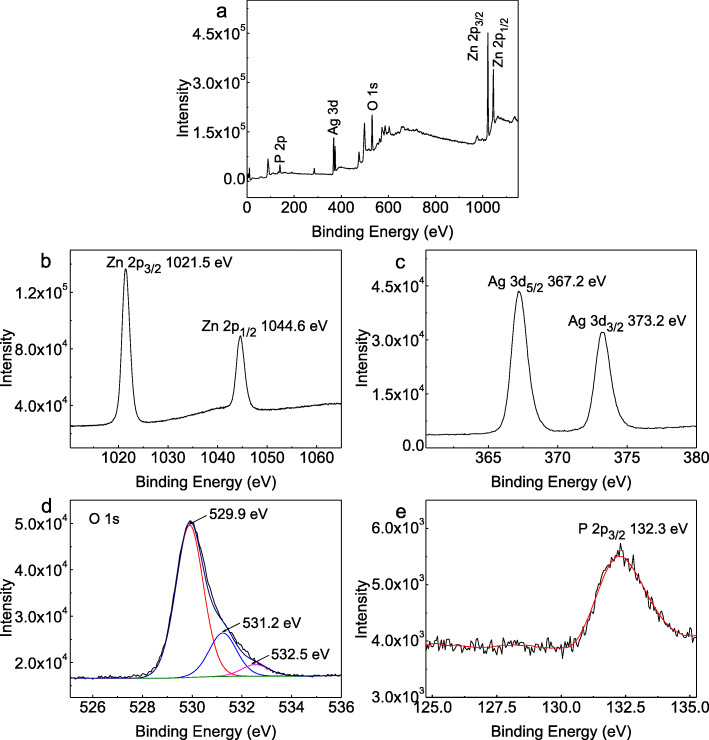
XPS spectra of Ag_3_PO_4_/T-ZnOw-2: **a** survey scan, **b** Zn 2p, **c** Ag 3d, **d** O1s, and **e** P 2p

XPS measurements were carried out to investigate the elemental composition and chemical states of the Ag_3_PO_4_/T-ZnOw-2 sample. Figure 3a exhibited the survey XPS spectrum and indicated the existence of the Zn, Ag, O, and P. Figure 3b showed the high-resolution XPS spectrum of the Zn 2p, and two binding energy peaks at 1021.5 and 1044.6 eV could be assigned to Zn 2p_3/2_ and Zn 2p_1/2_ of T-ZnOw, respectively [36]. Two peaks located at 367.2 and 373.2 eV could be attributed to Ag 3d_5/2_ and Ag 3d_3/2_ in the XPS spectrum of Ag 3d orbital (Fig. 3c), which was a characteristic of Ag^+^ [11]. As seen from the XPS spectrum of O 1s in Fig. 3d, there were three peaks at 529.9, 531.2, and 532.5 eV, which could be ascribed to the oxygen lattices in T-ZnOw [33], Ag_3_PO_4_ [37], and adsorbed –OH groups on the surface of Ag_3_PO_4_/T-ZnOw-2, respectively. A weak and broad band centered at 132.3 eV in Fig. 3 e could be ascribed to the characteristic P 2p from Ag_3_PO_4_ [38]. The XPS results further proved that Ag_3_PO_4_ and T-ZnOw had been compounded.

**Fig. 4:**
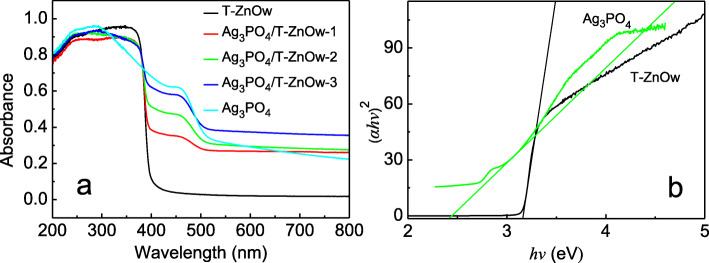
**a** UV-Vis DRS of T-ZnOw, Ag_3_PO_4_/T-ZnOw-1, Ag_3_PO_4_/T-ZnOw-2, Ag_3_PO_4_/T-ZnOw-3, and Ag_3_PO_4_. **b** Plots of (*αhv*)^2^ versus energy (*hv*)

UV-Vis diffuse reflectance spectra (DRS) were measured to study the optical absorption properties of the Ag_3_PO_4_/T-ZnOw heterostructures, together with those of T-ZnOw and Ag_3_PO_4_ (Fig. 4a). It could be observed that the absorption edge of T-ZnOw and Ag_3_PO_4_ was stated to be about 400 and 510 nm, respectively. Compared with T-ZnOw, the Ag_3_PO_4_/T-ZnOw heterostructures exhibited increasing absorption intensities in visible light region with the molar ratios of Ag_3_PO_4_ increasing. The widened absorption range and enhanced absorbance of the Ag_3_PO_4_/T-ZnOw heterostructures in the visible light region were benefit from the introduction of the narrower bandgap of Ag_3_PO_4_. The above results indicated that the Ag_3_PO_4_/T-ZnOw heterostructures were potential visible-light-driven photocatalysts. Furthermore, the bandgap energy of T-ZnOw and Ag_3_PO_4_ was evaluated by Kubelka-Munk function [39]. According to the plot of (*ahv*)^2^ versus energy, as shown in Fig. 4b, the bandgap value of T-ZnOw and Ag_3_PO_4_ was about 3.16 and 2.42 eV, respectively.

**Fig. 5:**
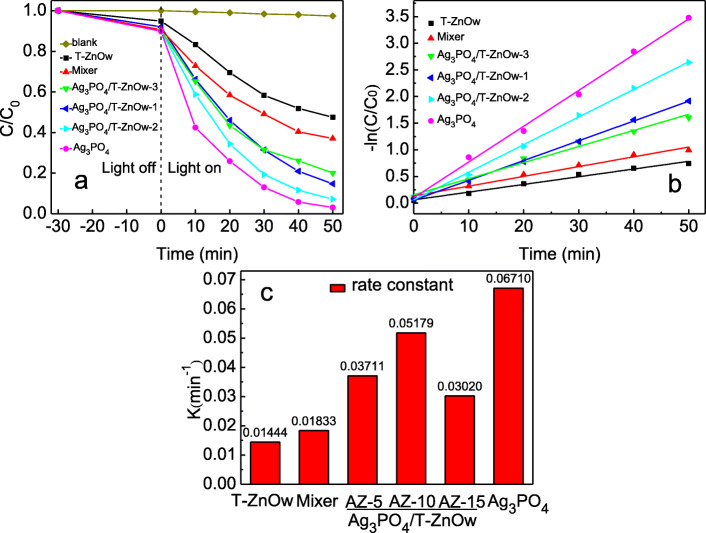
**a** Photodegradation of RhB with different photocatalysts. **b** The kinetic simulation curves. **c** Apparent rate constants

Photodegradation of RhB was used to evaluate the photocatalytic activity of T-ZnOw, Ag_3_PO_4_/T-ZnOw-1, Ag_3_PO_4_/T-ZnOw-2, Ag_3_PO_4_/T-ZnOw-3, Ag_3_PO_4_, and a mixture of T-ZnOw (26.41 mg) and Ag_3_PO_4_ (13.59 mg) under visible light. Figure 5a showed the photocatalytic activity of different samples for RhB degradation. After irradiation for 50 min, the degradation efficiency of T-ZnOw, Ag_3_PO_4_/T-ZnOw-1, Ag_3_PO_4_/T-ZnOw-2, Ag_3_PO_4_/T-ZnOw-3, Ag_3_PO_4_, and the mixture was 52.5%, 85.3%, 92.9%, 79.9%, 96.9%, and 62.9%, respectively. The physical mixture of T-ZnOw and Ag_3_PO_4_ which had the same composition proportion with Ag_3_PO_4_/T-ZnOw-2 displayed lower degradation efficiency of RhB than that of Ag_3_PO_4_/T-ZnOw-2, implying that Ag_3_PO_4_/T-ZnOw heterostructures were formed. With the molar ratios of Ag_3_PO_4_ increasing, the degradation efficiency of RhB was first increased and then decreased, and Ag_3_PO_4_/T-ZnOw-2 showed the highest degradation efficiency among the heterostructures, which was very closed to that of Ag_3_PO_4_. The agglomerated Ag_3_PO_4_ particles in the Ag_3_PO_4_/T-ZnOw-3 sample affected the size and the dispersion of Ag_3_PO_4_. It is well known that a smaller particle size decreases the electron-hole recombination possibility, thereby improving the photocatalytic performance of the material. In addition, the large size of Ag_3_PO_4_ particles in the Ag_3_PO_4_/T-ZnOw-3 sample may weaken the anchoring force between T-ZnOw and Ag_3_PO_4_ and destroy the heterojunction structure, which would limit the photocatalytic activity. The photodegradation of RhB followed the pseudo-first-order reaction, as shown in Fig. 5b. Figure 5c displayed the degradation rate constants of different photocatalysts, and the trend was the same as the degradation efficiency. The photodegradation rate constant of Ag_3_PO_4_/T-ZnOw-2 (0.05179 min^−1^) was 3.59 times that of T-ZnOw (0.01444 min^−1^). The above results clearly indicated that the photocatalytic activity of T-ZnOw was increased by Ag_3_PO_4_ modification. The improved photocatalytic activity of Ag_3_PO_4_/T-ZnOw heterostructures was benefited from the enhanced visible light absorbance intensity by loading Ag_3_PO_4_ on the surface of T-ZnOw, which would enable the Ag_3_PO_4_/T-ZnOw heterostructures to produce photogenerated carriers for the photodegradation of RhB under visible light. It should be noted that Ag_3_PO_4_ seemed to have the best photocatalytic activity among the as-prepared samples. Nevertheless, Ag_3_PO_4_ exhibited lower stability compared with Ag_3_PO_4_/T-ZnOw shown in the following discussion, which affected its long-term uses.

**Fig. 6:**
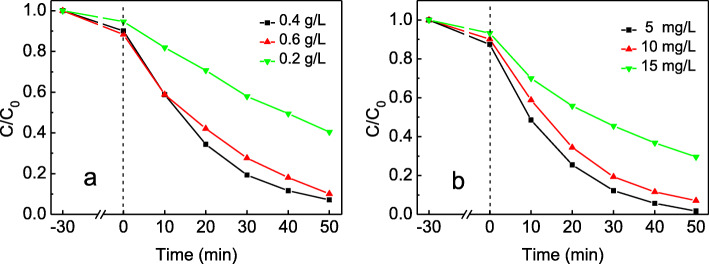
**a** Effect of different Ag_3_PO_4_/T-ZnOw-2 doses on the photodegradation of RhB. **b** Effect of different initial concentrations of RhB on the photocatalytic activity of Ag_3_PO_4_/T-ZnOw-2

Proper doses of photocatalyst in photodegradation system can reduce cost in economic viewpoint. Figure 6a showed the influence of the feed doses of Ag_3_PO_4_/T-ZnOw-2 on the degradation efficiency. The degradation efficiency obviously increased with the dose increased from 0.2 to 0.4 g/L and decreased thereafter. With the increasing of catalyst doses, the solution turbidity was increased and the light penetration into the reaction system was reduced at the same time. The lower visible light absorption of photocatalyst could decrease the degradation efficiency at a greater dose of the photocatalyst [40, 41].

The effect of different initial RhB concentrations on the photocatalytic activity of Ag_3_PO_4_/T-ZnOw-2 was studied and shown in Fig. 6b. When the initial concentrations were 5 mg/L, 10 mg/L, and 15 mg/L, the degradation efficiency of RhB were 98.2%, 92.9%, and 70.4%, respectively. The decrease in degradation efficiency may be due to the decrease of photons absorbed by the catalyst resulting from the increase in the path length of photons entering the solution with higher initial concentrations. Another reason may be more intermediates formed with the higher initial RhB concentrations which could form adsorption competition with initial reactants [42, 43]. However, too low initial concentration cannot fully show the photodegradation ability of the catalyst. Therefore, the initial concentration of RhB solution in the experiment was preferably 10 mg/L.

**Fig. 7:**
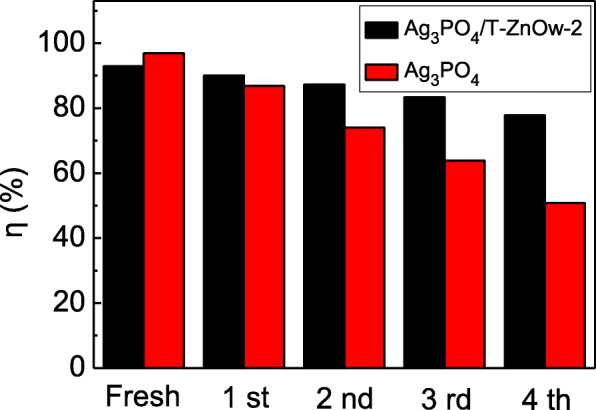
Four successive cycling runs for degradation of RhB over Ag_3_PO_4_/T-ZnOw-2 and Ag_3_PO_4_

The stability and reusability of a photocatalyst are crucial to measure its practical application [44]. It is well known that the Ag_3_PO_4_ photocatalyst can be easily reduced to Ag by photocorrosion, which limits its long-term practical application. Figure 7 displayed the recycling experiments for degradation of RhB over Ag_3_PO_4_/T-ZnOw-2 and Ag_3_PO_4_. After four successive cycles, the degradation efficiency of Ag_3_PO_4_ was obviously lower than that of Ag_3_PO_4_/T-ZnOw-2. The results presented above demonstrated that whereas Ag_3_PO_4_ photocatalyst showed a somewhat higher photocatalytic activity on first use, the Ag_3_PO_4_/T-ZnOw heterostructures appeared to be potential for long-term applications due to the enhanced stability. Pure Ag_3_PO_4_ photocatalyst is unstable if there is no sacrificial reagent added in the photocatalytic process [45]. The solubility of pure Ag_3_PO_4_ in aqueous solution is relatively high, which results in the decrease of its stability during the photocatalytic process [25]. Ag_3_PO_4_ can be reduced to metallic Ag by the photogenerated electrons, and a certain amount of Ag can form the structure of Ag/Ag_3_PO_4_/T-ZnOw. The further photocorrosion of Ag_3_PO_4_ in Ag/Ag_3_PO_4_/T-ZnOw composite can be inhibited by the transfer of electrons from the conduction band of Ag_3_PO_4_ to metallic Ag [46]. After Ag_3_PO_4_ particles were anchored on the T-ZnOw surfaces, Ag_3_PO_4_ particles and T-ZnOw had intimate contact with each other, and the smooth T-ZnOw surfaces served as an ideal refuge for Ag_3_PO_4_ and make less amount of Ag_3_PO_4_ stripping in aqueous solution, which was similar to the reported Ag_3_PO_4_/BiVO_4_ heterojunction [47]. Thus, Ag_3_PO_4_/T-ZnOw-2 heterostructure exhibited a good photocatalytic stability and possessed a degradation efficiency of 77.8% after recycling experiments.

**Fig. 8:**
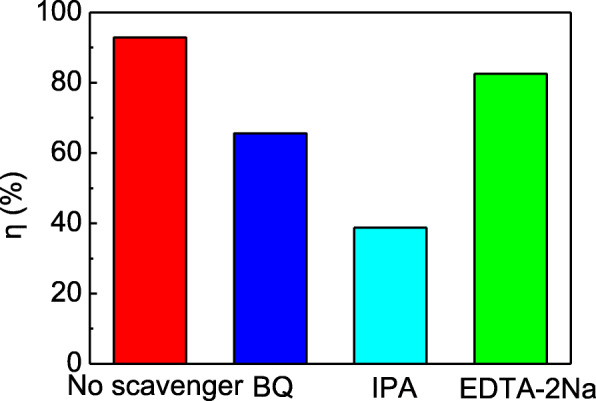
The influence on degradation efficiency of RhB by Ag_3_PO_4_/T-ZnOw-2 with different scavengers (scavenger dose = 0.2 mmol/L)

The effect of different scavengers on degradation efficiency of RhB by Ag_3_PO_4_/T-ZnOw-2 is shown in Fig. 8 after irradiation for 50 min. After the addition of IPA, BQ, and EDTA-2Na, the degradation efficiency diminished to 38.8%, 65.6%, and 82.6%, respectively, indicating that hydroxyl radicals (∙OH) and superoxide radicals (∙O_2_^−^) were the mainly active species, and holes (h^+^) played partially in the photocatalytic decoloration. The band position of Ag_3_PO_4_ and T-ZnOw was calculated by the following equation [18]:

$$ {\displaystyle \begin{array}{l}{E}_{\mathrm{VB}}=X-{E}^0+0.5{E}_{\mathrm{g}}\\ {}{E}_{\mathrm{CB}}={E}_{\mathrm{VB}}-{E}_{\mathrm{g}}\end{array}} $$

where *X* is the absolute electronegativity of the semiconductor and *E*_g_ is the bandgap energy. The *X* value for Ag_3_PO_4_ and ZnO are 6.16 [48] and 5.76 eV [49], respectively. According to the bandgap achieved in Fig. 4, the *E*_VB_ of Ag_3_PO_4_ and T-ZnOw was calculated to be 2.87 and 2.84 eV, and their homologous *E*_CB_ was 0.45 and − 0.32 eV, respectively.

**Scheme 1:**
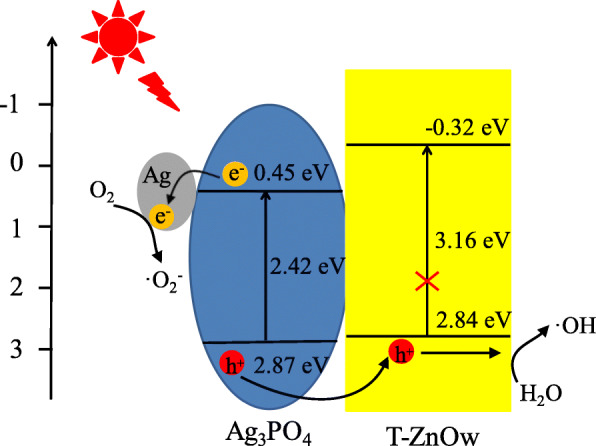
Schematic diagram of the possible photocatalytic mechanism of Ag_3_PO_4_/T-ZnOw

**Fig. 9:**
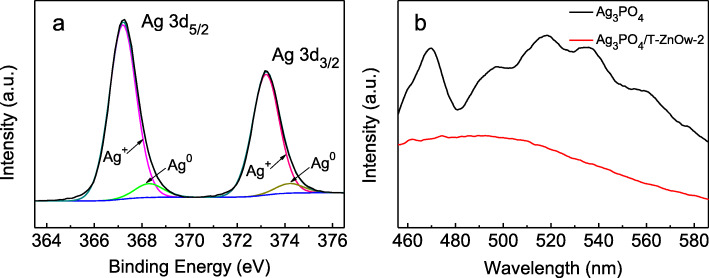
**a** Ag 3d XPS spectrum of Ag_3_PO_4_/T-ZnOw-2 sample after photocatalysis. **b** PL spectra of Ag_3_PO_4_ and Ag_3_PO_4_/T-ZnOw-2

The possible mechanism for the photocatalytic degradation of RhB could be proposed based on the above results, as shown in Scheme 1. The conduction band potential (CB − 0.32 eV) and valance band potential (VB 2.84 eV) of T-ZnOw were more negative than those of Ag_3_PO_4_ (CB 0.45 eV; VB 2.87 eV). The excited Ag_3_PO_4_ could produce electron-hole pairs under visible light illumination. Therefore, the photogenerated holes could shift from the VB of Ag_3_PO_4_ into the empty VB of T-ZnOw, which facilitated the effective separation of photogenerated electrons and holes. A part of photogenerated holes would react with the adsorbed H_2_O to form ∙OH as major active species, and the other part of holes adsorbed on the surface of the heterostructure could directly participate in the photodegradation of RhB. However, the CB potential of Ag_3_PO_4_ was 0.45 eV, which was higher than the reduction potential of O_2_/∙O_2_^−^ (− 0.33 eV) [29]. The photogenerated electrons on the conduction band of Ag_3_PO_4_ could not react with dissolved oxygen to form ∙O_2_^−^. A small amount of metallic Ag could be formed by the reaction between Ag^+^ from Ag_3_PO_4_ and photogenerated electrons by visible light illumination, which could be proved by the XPS spectrum of Ag_3_PO_4_/T-ZnOw-2 after illumination for 50 min in photocatalytic reaction. Figure 9a showed the Ag3d XPS spectrum of Ag_3_PO_4_/T-ZnOw-2 after photocatalysis for 50 min. The peak at 367.2 and 373.2 eV could be attributed to Ag^+^ ions, and the peak at 368.3 and 374.2 eV was assigned to the metallic Ag [11]. Then, photogenerated electrons on the conduction band of Ag_3_PO_4_ could transfer to metallic Ag, thus inhibiting the recombination of electron-hole pairs. Furthermore, the photogenerated electrons could be captured by dissolved oxygen to form ∙O_2_^−^, which played one of the major roles in the photodegradation of RhB. All of these photogenerated reactive species (∙OH, ∙O_2_^−^, and h^+^) could react with RhB to form CO_2_ and H_2_O and finally enhance the photocatalytic performance for degradation of RhB. Figure 9b presented the PL spectra of Ag_3_PO_4_ and Ag_3_PO_4_/T-ZnOw-2 with the excitation wavelength of 355 nm. Compared with pure Ag_3_PO_4_, the intensity of Ag_3_PO_4_/T-ZnOw-2 revealed a decrease in fluorescence, which was mainly attributed to the efficient charge carrier transfer between Ag_3_PO_4_ and T-ZnOw. The PL results were consistent with the proposed photocatalytic mechanism.

## Conclusions

In summary, Ag_3_PO_4_/T-ZnOw heterostructures were successfully fabricated by a facile in situ precipitation method. The Ag_3_PO_4_/T-ZnOw-2 catalyst exhibited superior photocatalytic activity for RhB degradation than pure T-ZnOw and possessed better stability and reusability compared with pure Ag_3_PO_4_. Under the optimum condition, Ag_3_PO_4_/T-ZnOw-2 showed the highest photocatalytic efficiency among the heterostructures and still possessed a degradation efficiency of 77.8% after four successive cycles. The efficient photocatalytic performance of Ag_3_PO_4_/T-ZnOw photocatalyst could be attributed to the enhanced visible light response. The Ag_3_PO_4_/T-ZnOw-2 photocatalyst also showed good stability. The investigation of the effect of different scavengers on degradation efficiency of RhB demonstrated that ∙OH and ∙O_2_^−^ were the mainly active species. A possible mechanism of the photodegradation pathway for RhB was proposed. Ag_3_PO_4_/T-ZnOw may be one of the potential photocatalysts for the use in the treatment of water pollutants.

## Data Availability

All data generated or analyzed during this study are included in this published article.

## Abbreviations

T-ZnOw: Tetrapod-like ZnO whisker
RhB: Rhodamine B
BQ: Benzoquinone
IPA: Isopropyl alcohol
EDTA-2Na: Ethylenediaminetetraacetic acid disodium salt
XRD: X-ray diffraction
SEM: Scanning electron microscopy
EDS: Energy dispersive X-ray spectroscopy
TEM: Transmission electron microscopy
HRTEM: High-resolution transmission electron microscopy
XPS: X-ray photoelectron spectroscopy
BEs: Binding energies
DRS: UV-Vis diffuse reflectance spectra
PL: Photoluminescence
